# GeneStoryTeller: a mobile app for quick and comprehensive information retrieval of human genes

**DOI:** 10.1093/database/bav048

**Published:** 2015-06-08

**Authors:** Stergiani V. Eleftheriou, Marilena M. Bourdakou, Emmanouil I. Athanasiadis, George M. Spyrou

**Affiliations:** ^1^Center of Systems Biology, Biomedical Research Foundation, Academy of Athens, Soranou Ephessiou 4, 115 27 Athens, Greece, and ^2^Department of Informatics and Telecommunications, University of Athens, 15784 Ilissia, Greece

## Abstract

In the last few years, mobile devices such as smartphones and tablets have become an integral part of everyday life, due to their software/hardware rapid development, as well as the increased portability they offer. Nevertheless, up to now, only few Apps have been developed in the field of bioinformatics, capable to perform fast and robust access to services. We have developed the GeneStoryTeller, a mobile application for Android platforms, where users are able to instantly retrieve information regarding any recorded human gene, derived from eight publicly available databases, as a summary story. Complementary information regarding gene–drugs interactions, functional annotation and disease associations for each selected gene is also provided in the gene story. The most challenging part during the development of the GeneStoryTeller was to keep balance between storing data locally within the app and obtaining the updated content dynamically via a network connection. This was accomplished with the implementation of an administrative site where data are curated and synchronized with the application requiring a minimum human intervention.

**Database URL**: http://bioserver-3.bioacademy.gr/Bioserver/GeneStoryTeller/.

## Introduction

With the advent of new methodologies in the field of bioinformatics, more and more online Web applications emerged, capable of handling large-scale data, having as a major goal to retrieve, handle, combine or compute data regarding every single gene, enlightening the understanding of its role from different perspectives. 

There is a wealth of data in the field of biology and we must have the appropriate repositories, tools and pipelines to exploit efficiently these data. Towards this direction, significant collections of data comprise specific repositories concerning gene and protein aspects like annotation, sequence, structure, functionality, interaction, networking as well as drug interactions and disease relation. A plethora of methods have been developed and implemented for examining the content of each database and extracting/identifying the crucial information. So far, this was performed by means of Web application tools, working on Windows/MAC/Linux Operating Systems (OS). The querying ability that the repositories offer, both in basic and in advanced mode, provides the user with an important knowledge discovery tool. When such tools are available via a mobile device even when there is no internet connection, then the usage of these repositories is always and from any location one click away, promoting and facilitating knowledge discovery.

In the new Era, smartphones’ and tablets’ (Android or Apple’s mobile operating system iOS) supporters are increasing in number day by day, since the combination of having lower mobility limitations with high performance devices is more feasible. However, despite the “blast” of mobile Apps, only few of them have been developed and proposed to the research community in the field of bioinformatics, namely the Hematopoietic Expression Viewer (1), YASARA View (2), DNAApp (3) and RCSB PDB Mobile (4). An application dedicated to search genes with primary information from the National Center for Biotechnology (NCBI) (http://www.ncbi.nlm.nih.gov/gene) is performed by the BioGene App (http://cbio.mskcc.org/tools/handheld-devices/index.html) supported by the Computational Biology Center at Memorial Sloan-Kettering Cancer Center. Nevertheless, BioGene, like all online applications, provides restrictions to users since internet access is mandatory and search is limited to one database (NCBI).

To overcome such limitations, we have developed a mobile application for Android platforms, named GeneStoryTeller, that enables smartphone/tablet users to instantly retrieve up-to-date information regarding any recorded human gene, in the form of a summary story with a robust and effective description. The main benefit of GeneStoryTeller App is that works offline and combines precompiled information from a list of eight well- established online data repositories.

## Materials and methods

Users of the GeneStoryTeller can search for any annotated human gene commonly referenced in biological publications and databases, and find information about its description summary, the drugs that are associated with as well as all its functional annotations and diseases. Furthermore, to exploit the advantages using a mobile app, we developed a personalized annotation functionality. Specifically, there is a separate table in the app’s database where the personal annotations regarding the gene query results can be saved along with a time stamp. These annotations can be retrieved by the user for further processing or querying. The annotation functionality can be recalled by pressing the star icon on the upright corner of the main navigation screen. The yellow and grey colours on the star indicate the potentiality of adding a new annotation and of indexing the existing annotations, respectively.

**Figure 1.**
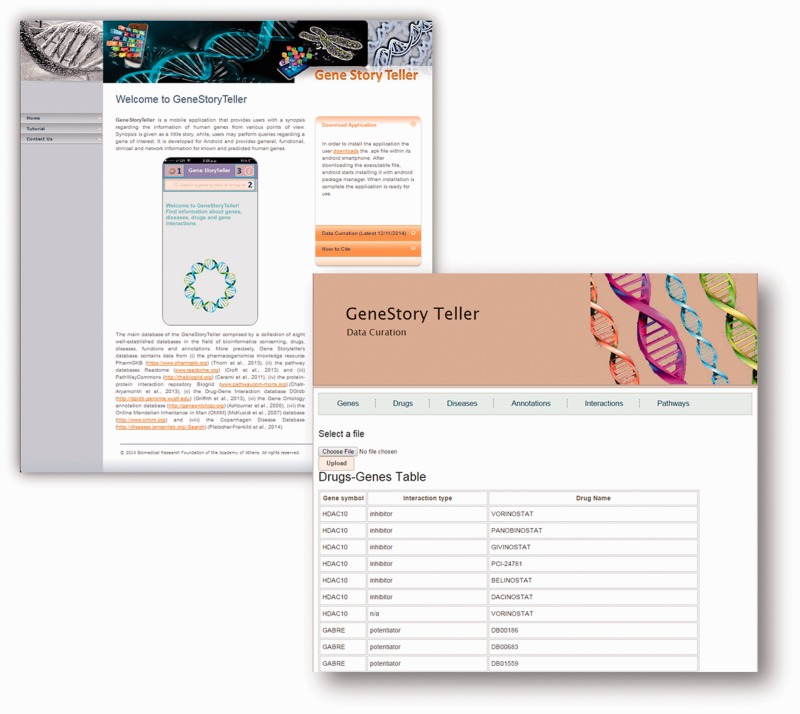
(**A**) Main web application page can be found on http://bioserver-3.bioacademy.gr/Bioserver/GeneStoryTeller/. Users are able to find information regarding the installation process, as well as a tutorial page with an example usage pipeline. (**B**) Administrative curation page where update can be performed for each category (Genes, Drugs, Diseases, Annotation, Interaction and Pathways).

The Titanium Studio from Appcelerator (http://www.appcelerator.com/) was used to construct the entire App. This software is a cross-compiled development tool, that uses internet technologies (i.e. html, css, javascript), joining javascript with system libraries. Specifically, the main code of the App is written in javascript, while css is used for the graphical design. A run time environment that is included in the tool undertakes the compilation of the code. A web page that hosts the installation file, as well as supplementary information regarding the application can be found on http://bioserver-3.bioacademy.gr/Bioserver/GeneStoryTeller/ (Figure 1A).

## GeneStoryTeller: curation process

The main challenge while developing the GeneStoryTeller was the construction of a robust synchronization mechanism for the continuous curation of the database. At the same time, keeping balance between storing content locally on the device and obtaining curated data dynamically through the use of network connection was not a trivial task during the development stage of the App. To cope with these difficulties, a supplementary web database was created (see Figure 1B). Through the use of this administrative site, new data gathered from different biological databases can be uploaded to the server. To resolve any compatibility issues arisen due to the diversity of existing biological databases, input data are firstly transformed into manageable file formats, such as Comma Separated Value (CSV) or Gene Matrix Transposed file (GMT) and then uploaded to the server. Synchronization is then accomplished through predefined queries in Structured Query Language (SQL).

The supplementary web database in the administrative site acts as a central repository of precompiled information and data, providing the app only with the necessary precompiled data through the synchronization mechanism. In order to avoid double entries between updating and existing data that are already up to date, a time stamp is used in the local database that is checked before each update. The uncompressed local database needs about 120 MB of storage and even if the disease associations, drug interactions and other information present a major increase their content, given that all the data are textual, it would be affordable by any modern device local storage.

The main database of the GeneStoryTeller comprises a collection of eight well-established databases in the field of bioinformatics concerning, drugs, diseases, functions and annotations, as listed in Table 1. More precisely, Gene Storyteller’s database contains data from (i) the pharmacogenomics knowledge resource PharmGKB (https://www.pharmgkb.org/) (5), (ii) the pathway databases Reactome (www.reactome.org/) (6) and (iii) PathWayCommons (www.pathwaycom-mons.org/) (7), (iv) the protein–protein interaction repository Biogrid (http://thebiogrid.org/) (8), (v) the Drug–Gene Interaction database DGIdb (http://dgidb.genome.wustl.edu/) (9), (vi) the Gene Ontology annotation database (http://geneontology.org/) (10), (vii) the Online Mendelian Inheritance in Man (OMIM) (11) database (http://www.omim.org/) and (viii) the Copenhagen Disease Database (http://diseases.jensenlab.org/-Search) (12).[Table bav048-T1]

**Table 1. bav048-T1:** Detailed list of eight well-established web databases comprising the backbone of the GeneStoryTeller

AA	Web database	Database home page
1	PharmGKB	https://www.pharmgkb.org/
2	Reactome	http://www.reactome.org/
3	PathWayCommons	http://www.pathwaycom-mons.org/
4	Biogrid	http://thebiogrid.org/
5	DGIdb	http://dgidb.genome.wustl.edu/
6	Gene Ontology	http://geneontology.org/
7	Online Mendelian Inheritance in Man OMIM	http://www.omim.org/
8	Copenhagen Disease Database	http://diseases.jensenlab.org/-Search

The first column indicates the database name, while the second column describes the link of the home page.

## GeneStoryTeller: usage

**Figure 2.**
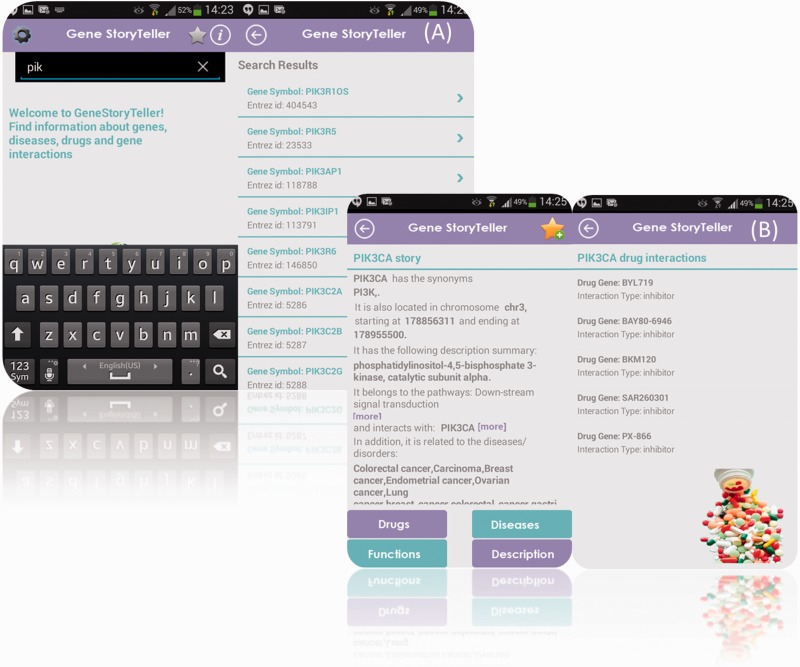
(**A**) Main search engine navigation screen of the GeneStoryTeller App. In this example, the user types initials ‘pik’ and the searching engine has returned the corresponding matching gene symbols. (**B**) Generated navigation panel of the resulted description summary story. In this example, the user has selected PIK3CA gene and the corresponding story is presented on the left part of the image. On the right part of the image, there is an example of the drug interactions of the selected PIK3CA.

To operate GeneStoryTeller, a user opens the App and searches for a desired gene in either Gene Symbol or Entrez ID format using the search box at the top of the searching screen (see Figure 2A). Once the gene has been selected, all the available information concerning the corresponding gene is presented in the form of a story (see Figure 2B). This story includes information about gene's synonyms, chromosome location, start and stop position inside the chromosome, as well as its description summary. Complementary information concerning details about its pathways, interactions with other genes, diseases that are related to the specific gene, drug-genes interactions and functional annotations are also available, if applicable. Besides the main story text, there are also four shortcut buttons in the bottom of the screen where access to drugs, diseases, functions and description of the selected gene is also provided.

Apart from the searching navigation panel, user can navigate to synchronization screen, where synchronization may be performed. There are two options for this, the auto and the manual synchronization process. This process imposes internet access so as to start a manual synchronization, or set the time for the auto synchs among one, 3 or 6 months. There is also a selection to disable synchronization process and enable it manually in due time.

## Conclusion

We present here a novel user-friendly smartphone application, the GeneStoryTeller that presents latest information regarding genes in a form of a summary story. This information is updated in regular time intervals by compiling latest data gathered from eight well-established gene annotation repositories. GeneStoryTeller works offline with no restrictions and internet connection is required only if a new GeneStoryTeller update is available.

## Funding

NSRF 2007–2013, co-funded by the European Regional Development Fund and national resources, under grant “Cooperation” [No. 09ΣYN-11-675] to M.B., E.A. and G.S. The funders had no role in study design, data collection and analysis, decision to publish, or preparation of the manuscript. Funding for open access charge: Biomedical Research Foundation of the Academy of Athens.

*Conflict of interest*. None declared.
